# Oral microbiota–driven immune modulation along the oral–gut axis: from local signals to systemic inflammation

**DOI:** 10.1038/s41522-026-00912-0

**Published:** 2026-01-10

**Authors:** Chunhao Li, Yue Fan, Xingming Chen

**Affiliations:** Department of Otolaryngology-Head and Neck Surgery, Peking Union Medical College Hospital, Peking Union Medical College and Chinese Academy of Medical Sciences, Beijing, China

**Keywords:** Immunology, Microbiology

## Abstract

The oral–gut axis is a key pathway through which oral microbiota modulate systemic immunity. Oral bacteria and their derivatives, including microbial-associated molecular patterns and extracellular vesicles, can translocate to the gut, evade mucosal defenses, interact with local immune cells, and disrupt epithelial integrity. This review highlights mechanisms of gut colonization, immune modulation via pattern recognition receptors, and contributions to distal organ inflammation, providing a framework for understanding microbiota-driven systemic diseases.

## Introduction

The oral–gut axis constitutes a pivotal route through which oral microbiota and their components modulate the gastrointestinal and systemic immune landscapes^1^. Through swallowing, circulatory dissemination, immune cell trafficking, and the release of bacterial extracellular vesicles (BEVs)^2^, components of oral bacteria, including whole cells, DNA, and lipopolysaccharides (LPS), can reach the gastrointestinal tract^3^. Under permissive or pathological conditions, these microbial elements may penetrate mucosal barriers^4^, allowing them to interact directly with intestinal epithelial cells (IECs), lamina propria immune cells, and mesenteric lymphoid tissues. This interaction reshapes mucosal immune homeostasis and initiates systemic inflammatory cascades^5^.

Recent studies have revealed strong links between the oral microbiome and chronic diseases affecting distal organs, including metabolic, immune-mediated, and neurodegenerative conditions. Among the proposed mechanisms, the oral–gut axis not only represents a physical and physiological bridge between the oral cavity and the gastrointestinal tract but also embodies the complexity of interregional microbial crosstalk and immune integration. After translocating to the gut, oral microbes can alter colonization patterns, disrupt microbial metabolism, and activate mucosal immune sensors, such as toll-like receptors (TLRs), thereby promoting both local and systemic immune activation^6^. For example, pathogenic oral bacteria have been shown to disrupt gut microbial ecology, compromise epithelial barrier function, and engage mucosal immune sensors such as TLRs, collectively leading to local inflammation and immune activation in distal organs^7,8^. These processes are increasingly recognized in the pathogenesis of diseases such as metabolic dysfunction–associated steatotic liver disease (MASLD), Alzheimer’s disease (AD), and Parkinson’s disease (PD)^9^. Although investigations into oral microbial translocation and its systemic sequelae are still at an early stage, the oral–gut axis is now recognized as a critical conduit mediating cross-organ microbial and immune interactions.

This review aims to elucidate the mechanisms through which oral microbiota influence systemic immune responses via the gut, focusing on their migration routes, colonization potential, immunomodulatory functions, and organ-specific consequences, ultimately informing novel strategies for the prevention and treatment of chronic inflammatory disorders.

## Oral microbial migration and entry into the gut: pathways and invasion mechanisms

### Migration routes and risk factors

#### Oral microbes and gut colonization in healthy individuals

In healthy individuals, a series of physical and immune barriers, including salivary antimicrobial defenses^10^, gastric acid sterilization^11^, bile salt–mediated inhibition^12^, the intestinal mucus layer^13^, and epithelial tight junctions^14^, jointly provide robust protection against cross-regional microbial translocation. A metagenomic analysis by Cheung et al. involving 470 healthy Chinese adults demonstrated marked compositional differences between oral and gut microbiota, with minimal oral microbial contribution to the gut ecosystem (bacterial taxa ≤0.05%, fungal taxa ≤5%)^15^. Similarly, Etienne-Mesmin et al.^10^. showed using in vitro gut simulations that artificial inoculation with oral microbiota did not appreciably alter the microbial composition of the gut lumen or mucosal compartments. These observations suggest that in the presence of intact mucosal barriers, oral microbes are unlikely to breach host defenses or establish stable colonization within the gut. Nevertheless, approximately 1.5 l of saliva are produced daily, transporting billions of oral microbes into the digestive tract. Metagenomic profiling has revealed that approximately 45% of gut bacterial species share phylogenetic overlap with oral taxa^16^, and fecal samples from healthy individuals often harbor oral commensals such as *Streptococcus*, *Veillonella*, *Actinomyces*, and *Prevotella*^2,3^. However, microbial detection does not equate to colonization. Whether oral microbes can truly colonize the gut remains controversial: (1) some shared species may be co-habitants established during early-life colonization^17^; (2) many “oral-origin” species detected in the gut may merely be transient passengers, lacking the capacity for sustained adhesion, proliferation, or ecological competitiveness^10^; (3) conventional 16S rRNA and metagenomic approaches cannot differentiate viable bacteria from residual DNA, nor can they localize microbial presence to specific niches (e.g., mucosa-attached vs. lumen-dispersed regions).

Thus, while some oral microbes may reach the gut via swallowing, their transient presence, constrained by intact barriers and ecological competition, makes stable colonization and functional impact unlikely in healthy individuals. Whether such microbes can adhere and proliferate within the gut microenvironment remains to be fully elucidated.

#### Mechanisms of microbial translocation

By contrast, a range of pathological conditions can compromise the integrity of the oral–gut barrier, substantially elevating the risk of translocation and colonization by oral-derived microbes^18^. Several host- and environment-related susceptibility factors have been identified: (1) Host-associated factors, including aging, hyposalivation, and poor oral hygiene (e.g., plaque accumulation), can impair mucosal defenses, thereby facilitating microbial translocation. Fecal samples from individuals with these conditions show marked enrichment of oral-origin taxa^19^; (2) Pharmacological interventions: antibiotics downregulate the expression of key barrier proteins, such as mucin 2 (MUC2), zonula occludens-1 (ZO-1), and occludin^20,21^, disrupting the epithelial barrier and thinning the mucus layer^22^, thereby increasing the risk of bacterial translocation. Additionally, antibiotic-induced dysbiosis^23^ may foster the overgrowth and intestinal colonization of oral pathogens such as *Streptococcus* and *Porphyromonas gingivalis* (*P. gingivalis*). Proton pump inhibitors (PPIs) reduce gastric acidity by inhibiting acid secretion, thereby increasing gastric pH and promoting the survival of oral microbes (e.g., *Rothia*, *Streptococcus*) during passage through the gastrointestinal tract^24,25^; (3) Infectious conditions: chronic infections such as periodontitis can generate persistent microbial load in the oral cavity. Pathogens like *P. gingivalis* and *Klebsiella* spp. may enter the gut via swallowing or hematogenous routes, contributing to dysbiosis and intestinal inflammation^26^; (4) Mechanical interventions or trauma, including tooth brushing, mastication, or dental procedures such as extraction, implantation, or orthodontics, can induce transient bacteremia, allowing oral microbes to enter systemic circulation^27^. Collectively, these host factors, pharmacological treatments, and infectious states can synergistically compromise mucosal barrier integrity, markedly increasing the likelihood of oral-gut microbial translocation and downstream systemic consequences.

Beyond the bacteria themselves, BEVs have emerged as key mediators of inter-tissue microbial communication. BEV is the general term for vesicles released by bacteria and encompasses outer membrane vesicles^28^ (OMVs) from Gram-negative bacteria, cytoplasmic membrane vesicles (CMVs) from Gram-positive bacteria, as well as other subtypes such as outer–inner membrane vesicles (OIMVs) and explosive outer-membrane vesicles (EOMVs), reflecting distinct biogenesis pathways. Some studies have incorrectly applied the term OMV to vesicles from Gram-positive bacteria; therefore, the use of the overarching term BEV is recommended when multiple bacterial types are involved or when vesicle biogenesis is unclear^29^. BEVs are nanoscale vesicles (20–400 nm in diameter) enriched with proteins, lipids, microRNAs, and inflammatory mediators^30^, characterized by high structural stability^31^ and notable penetrability. They can breach epithelial barriers of the oral and gastrointestinal mucosa through both transcellular pathways (e.g., endocytosis) and paracellular routes (e.g., disruption of tight junctions)^32^. Experimental models have demonstrated that gingival exposure to *P. gingivalis*-derived BEVs (strain ATCC 33277) elevates circulating levels of LPS and tumor necrosis factor alpha (TNF-α), culminating in colonic inflammation and microbial dysbiosis^33^. These vesicles exert potent pro-inflammatory effects by compromising the intestinal barrier and promoting M1 macrophage polarization, thereby amplifying the production of pro-inflammatory cytokines^34^. Salivary BEVs have also been shown to aggravate dextran sulfate sodium induced colitis in murine models^35^.

In addition, BEVs from *Fusobacterium nucleatum* (*F. nucleatum*) can trigger IEC death through activation of the receptor-interacting serine/threonine-protein kinase 1 (RIPK1)-dependent signaling cascade^36^, a key pathway mediating programmed cell death and inflammatory responses^37^.

Moreover, BEVs may thus act as “nano-containers” for inflammatory signaling, facilitating microbial cross-talk and functional modulation without the need for live bacterial migration. *F. nucleatum*-derived BEVs containing the adhesin Fimbriae-associated adhesin A (FadA) can translocate to distal sites, such as joints, and trigger local inflammatory responses, exemplifying BEVs’ role in mediating systemic pathogenic effects^38^.

In addition to direct microbial translocation and BEV-mediated mechanisms, certain oral bacteria can hijack host immune cells to enable long-range dissemination, a strategy akin to a “Trojan horse.” *P. gingivalis*, an exemplar oral pathogen capable of reshaping the microbial community, is a well-characterized example of this immune-cell hijacking strategy^39^. Strains expressing short fimbriae (Mfa1⁺) can bind the C-type lectin receptor dendritic cell-specific ICAM-grabbing non-integrin (DC-SIGN)—a receptor on myeloid dendritic cells that mediates the recognition and uptake of pathogens and self-antigens^40^—on conventional dendritic cells (cDCs), enabling encapsulation within single-membrane compartments that evade autophagic clearance and lysosomal degradation, thereby promoting intracellular persistence^41^. In vitro studies demonstrate that cDCs infected with *P. gingivalis* strains (Pg381 and PgDPG-3) display an immature phenotype, impaired differentiation, upregulated pro-inflammatory cytokines (e.g., interleukin-1 beta [IL-1β], interleukin-6 [IL-6]), and increased expression of matrix metalloproteinase-9 (MMP-9), collectively enhancing their chemotactic and migratory potential^42^. Clinically, *P. gingivalis*-harboring cDCs have been detected in the peripheral blood of patients with chronic periodontitis, with co-localization observed in atherosclerotic lesions^42^. These findings suggest that *P. gingivalis* exploits infected peripheral immune cells for systemic dissemination, potentially reaching distal sites such as the gastrointestinal tract.

In summary, *P. gingivalis* infects circulating cDCs, evades intracellular degradation, reprograms their immune phenotype, and enhances their migratory behavior, together constituting a plausible “Trojan horse” mechanism for microbial translocation along the oral–gut axis.

### Colonization capacity of oral bacteria in the gut and its determinants

#### Definition and challenges of ectopic colonization

Determining the extent to which oral bacteria truly colonize the gut presents two major challenges. Firstly, identifying whether gut-detected microbes originate from the oral cavity; secondly, distinguishing colonization from transient passage or external contamination. Current methodologies primarily rely on metagenomic or high-resolution 16S rRNA sequencing to infer oral microbial presence, using cross-sample taxonomic comparison, ecological trait analyses, and spatial abundance profiling. However, due to the coexistence of some microbial taxa across multiple mucosal surfaces, it remains difficult to determine whether the presence of certain bacteria in the gut represents true oral translocation or native colonization. For instance, taxa such as *Streptococcus*^19^, *Veillonella*^43^, and *Actinomyces*^19^, which are frequently classified as oral commensals, have been detected in the guts of healthy individuals, potentially derived from early-life colonization, vertical transmission, or lifestyle-associated microbiota shaping. Therefore, one-time detection of these species in fecal samples is insufficient to confirm oral-to-gut translocation. Moreover, assessments based solely on relative abundance or detection frequency lack directional resolution in the context of shared taxa. For example, even if a species is abundant in the oral cavity but present at low levels in the gut, this gradient alone cannot confirm directional migration without supporting data.

To overcome these limitations, some studies compare paired oral and fecal samples from the same individuals to identify amplicon sequence variants (ASVs) or strains with oral-dominant abundance that are simultaneously detected in stool—particularly if such taxa are uncommon across other individuals. Representative oral taxa such as *Haemophilus parainfluenzae, Streptococcus salivarius, Streptococcus mitis, and Streptococcus parasanguinis* have been identified with this distribution pattern, suggesting possible oral origin and inter-site mobility^44^.

Longitudinal studies further enhance this inference by assessing whether a particular oral ASV exhibits persistence or expansion within the gut microbiota over time^45^. Additional methods include strain-level single-nucleotide polymorphism tracing^46,47^ and functional transcriptomics^48,49^, which allow evaluation of whether these bacteria can adapt, proliferate, and interact within the gut environment. Phylogenetic clustering that links a gut-detected strain more closely to oral rather than intestinal lineages provides further evidence supporting oral-to-gut transmission.

Taken together, confirmation of oral bacterial colonization in the gut requires integration of ecological, genomic, and functional evidence; species-level detection alone is inadequate. Future work should incorporate high-resolution strain-tracking tools, spatial sampling, and in situ activity profiling to enhance precision in assessing ectopic colonization.

#### Cross-regional colonization capacity of oral bacteria

**Fig. 1:**
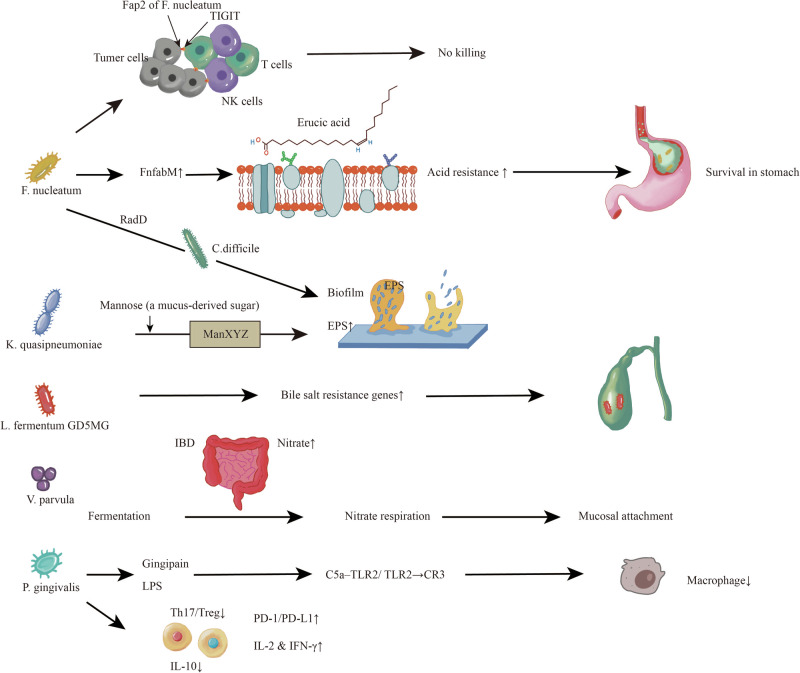
Mechanisms of oral bacterial translocation and gut colonization. Oral bacteria (e.g., *Fusobacterium nucleatum*, *Porphyromonas gingivalis*, *Veillonella parvula*) translocate to the gut via swallowing, where they overcome gastrointestinal barriers through enhanced acid resistance and bile tolerance. In the gut environment, they adapt by utilizing mucus-derived mannose, engaging in synergistic colonization (e.g., *F. nucleatum* RadD adhesin binding *Clostridioides difficile*), and switching metabolic modes (e.g., *V. parvula* switching from fermentation to nitrate respiration under IBD-associated nitrate-rich conditions). These bacteria evade host immunity by: inhibiting innate responses (*F. nucleatum* Fap2 binding TIGIT on NK/T cells → blocked cytotoxicity; P. gingivalis LPS/gingipains disrupting C5a–TLR2/ TLR2 → CR3 signaling → phagocytosis suppression), reprogramming adaptive immunity (PD-1/PD-L1↑ → suppressed IL-2/IFN-γ; Treg/IL-10↑ → immune tolerance). Collectively, microbial traits, ecological adaptation, and immune modulation converge to enable long-term mucosal colonization. TIGIT T cell immunoreceptor with Ig and ITIM domains, NK natural killer cells, LPS lipopolysaccharide, C5a complement component 5a, CR3 complement receptor 3, TLR2 toll-like receptor 2, PD-1 programmed cell death protein 1, PD-L1 programmed death-ligand 1, IL-2 interleukin-2, IFN-γ interferon-gamma, Treg regulatory T cell, IL-10 interleukin-10.

Only a subset of oral bacteria that reach the gut are capable of stable colonization. Colonization capacity varies substantially across genera and strains, influenced by physiological resilience, metabolic plasticity, and microbial interactions (Fig. 1).

First, to overcome physicochemical barriers, some strains upregulate membrane-stabilizing genes or stress-response pathways. For example, *F. nucleatum* demonstrates strong acid tolerance in simulated gastric environments via *F. nucleatum* fabM (*FnfabM*)-mediated erucic acid (a long-chain monounsaturated fatty acid) synthesis^50^, while *Lactobacillus fermentum* GD5MG harbors multiple bile resistance genes that support its persistence in the small intestine^51^. Second, certain oral bacteria can utilize gut-specific nutrients, conferring a competitive advantage within local ecological niches. For example, Suguru Miki et al.^52^ aimed to investigate how oral-derived microorganisms may promote intestinal mucosal colonization through the mannose phosphate transport system (ManXYZ). Although the *Klebsiella quasipneumoniae* (*K. quasipneumoniae*) strain used in their study (ATCC 700603) has not been confirmed as an oral isolate, the findings demonstrated that *K. quasipneumoniae* can utilize the ManXYZ system to efficiently uptake mucosal mannose, thereby promoting extracellular polysaccharide (EPS) synthesis and stable biofilm formation, which enhance adhesion and colonization capacity. Third, oral bacteria may co-aggregate with fellow microbes or establish symbiotic networks with gut residents. For example, *F. nucleatum* interacts with *Clostridioides* spp. via RadD adhesin to form mucus-layer biofilms^53^, while subspecies such as *F. nucleatum* C2 animalis and *polymorphum* exhibit niche-forming traits including iron acquisition and adhesion gene expression^54^.

In essence, oral bacterial colonization is not incidental but reflects adaptive traits selected under intestinal ecological pressures.

#### Host immune tolerance and microbial adaptation mechanisms

The persistence of oral bacteria in the gut hinges not only on ecological competition but also on their ability to evade host immune responses. Certain strains employ specialized strategies to modulate host immune defenses. For instance, the Fap2 protein of *F. nucleatum* strains (e.g., ATCC 23726, ATCC 49256, CTI-2) directly binds to the inhibitory receptor T cell immunoreceptor with Ig and ITIM domains on natural killer (NK) cells and T cells, suppressing cytotoxicity and dampening immune surveillance^55^.

*P.gingivalis*, another well-recognized oral pathobiont, has evolved multiple strategies to subvert innate and adaptive defenses. Its atypical LPS antagonizes TLR4; in contrast, it does not directly antagonize TLR2, but instead exploits TLR2 crosstalk with other receptors to subvert host defense. Gingipains (HRgpA, RgpB) exploit C5a–TLR2 crosstalk to impair microbicidal activity, and fimbriae engage CR3 via TLR2 signaling to promote intracellular persistence in macrophages^56^.

It also modulates adaptive immunity by skewing the balance between T helper (Th) 17 cells^57^ (interleukin-17-producing helper T cells) and regulatory T cells^58^ (Tregs, immune cells that suppress excessive immune activation), upregulating programmed death-1^59^ (PD-1, an inhibitory receptor on cells)/programmed death-ligand 1 (PD-L1, its ligand) signaling, and inhibiting the expression of interleukin-2^60^ (IL-2, a T cell growth factor) and interferon-gamma^61^ (IFN-γ, an important regulator of host defense system by mediating both innate and adaptive immune responses)^62^. In addition, wild-type *P. gingivalis* ATCC 33277 (FimA-intact) has been shown to promote Treg induction and interleukin-10 (IL-10) secretion, thereby facilitating long-term, low-abundance mucosal colonization^63,64^. Meanwhile, colonizing oral microbes dynamically adjust their metabolic programs and surface structures in response to intestinal pressures. For example, *Veillonella parvula* adapts to inflammatory niches in patients with inflammatory bowel disease (IBD) by switching to nitrate respiration, using nitrate as a terminal electron acceptor to maintain energy metabolism and colonization^65^.

These findings highlight the bidirectional adaptation between oral microbes and the host immune system by which pathogens evade recognition while shaping local immune tolerance.

## Gut immune reprogramming by oral microbiota

**Fig. 2:**
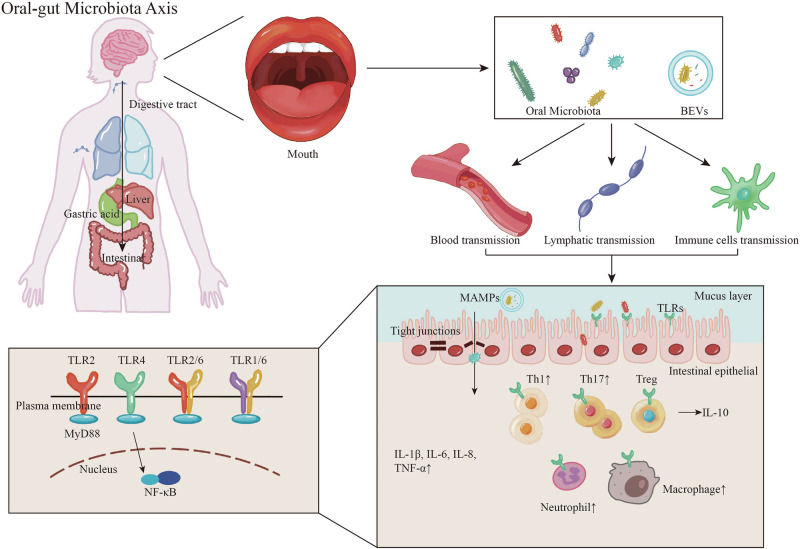
Mechanisms of the oral–gut microbiota axis in mucosal and systemic immune activation. Oral microbiota and their extracellular vesicles can enter the gastrointestinal tract through swallowing, and under pathological conditions, may also disseminate via the bloodstream, lymphatic circulation, or immune cell trafficking. These microbial components—including whole bacteria, MAMPs, and BEVs—interact with the intestinal mucosal barrier, leading to tight junction disruption and activation of PRRs such as TLR2, TLR4, TLR2/6, and TLR1/6. These pathways converge on MyD88-NF-κB signaling, promoting Th1 and Th17 polarization, recruitment of neutrophils and macrophages, and secretion of proinflammatory cytokines (e.g., IL-1β, IL-6, IL-8, TNF-α), while suppressing regulatory T cells (Tregs) and IL-10 production. These immune alterations contribute to local mucosal inflammation and may propagate systemic immune responses affecting distal organs such as the liver, lungs, and brain. BEVs bacterial extracellular vesicles, IL-1β interleukin-1 beta, IL-6 interleukin-6, IL-8 interleukin-8, IL-10 interleukin-10, MAMPs microbe-associated molecular patterns, MyD88 myeloid differentiation primary response 88, NF-κB nuclear factor kappa B, PRRs pattern recognition receptors, Th1 T helper 1, Th17 T helper 17, TLR1/6 toll-like receptor 1/6 heterodimer, TLR2 toll-like receptor 2, TLR2/6 toll-like receptor 2/6 heterodimer, TLR4 toll-like receptor 4, TNF-α tumor necrosis factor alpha, Tregs regulatory T cells.

Although the stable colonization of oral bacteria in the gut remains debated, accumulating evidence suggests that translocated oral taxa can actively interact with the intestinal immune milieu. These bacteria are not in a “silent” state but engage in complex crosstalk with the host mucosal immune system, influencing both local and systemic immune homeostasis. This interaction profoundly shapes the intestinal immune landscape, promoting infiltration of specific myeloid cell subsets, eliciting proinflammatory signatures, and triggering immune responses that may favor tumor progression^66–68^. Such crosstalk is mediated via microbial-associated molecular patterns (MAMPs), secreted metabolites, and structural components, initiating both innate and adaptive immune responses. Consequent effects include modulation of immune cell polarization, alteration of epithelial barrier integrity, and reshaping of local inflammatory landscapes. Moreover, sustained host-microbe interactions may imprint mucosal “inflammatory memory”, laying the groundwork for persistent immune dysregulation in chronic disease contexts (Fig. 2).

Both the oral and intestinal microbiota interact with host immunity through conserved pattern recognition receptors (PRRs), which are pivotal for detecting microbial signals, orchestrating immune responses, and preserving epithelial homeostasis. Among them, TLRs, a major subclass of membrane-bound PRRs, are the most prominent family members^57^. TLRs recognize conserved microbial structures, including LPS, lipoproteins, peptidoglycans, and flagellin^69–71^. Upon activation, they initiate downstream signaling cascades involving NF-κB and MAPK^72,73^, regulating the expression of pro-inflammatory cytokines^74,75^ and antimicrobial peptides (AMPs), thereby contributing to both defense and tolerance at mucosal sites^76–78^.

In the oral cavity, TLRs are widely expressed on keratinized squamous epithelial cells^79^ and local innate immune cells^80,81^. Notably, TLR2 and TLR4 have been implicated in periodontitis, where they signal via myeloid differentiation primary response 88 (MyD88)-dependent pathways to drive IL-1β, TNF-α, and other pro-inflammatory mediators, contributing to immune dysregulation and tissue destruction^82,83^.

In parallel, IECs also express a range of PRRs capable of recognizing MAMPs, regardless of their microbial origin^84^. Thus, oral bacteria that translocate to the gut can still be sensed by these receptors, with their components—such as LPS and flagellin—activating gut mucosal immunity. For example, flagellin and outer membrane proteins (e.g., OmpX) from oral *Klebsiella* spp. with a flagellar assembly system (such as Ka-11E12, but not typical non-flagellated strains) can be detected by TLR4 signaling through MyD88, leading to activation of dendritic and glial cells and promoting Th1 responses in the gut mucosa^85^. Similarly, *P. gingivalis* and its LPS activate gut immunity primarily via TLR4, with partial contribution from TLR2, disrupting the Th17/Treg balance, inducing local inflammation, and impairing epithelial barrier function^86^. Conversely, beneficial oral commensals such as *Lactobacillus acidophilus* enhance tight junction integrity via TLR2/TLR1 and TLR2/TLR6 heterodimers, thereby supporting mucosal barrier homeostasis^87^.

Importantly, individual TLR subtypes exert distinct and sometimes opposing effects in the gut. TLR2, particularly in the context of probiotic-rich environments (e.g., *Lactobacillus* and *Bifidobacterium*), is associated with improved intestinal barrier integrity, balanced immune responses, and epithelial resilience^88^. In contrast, TLR4—primarily responsive to LPS from Gram-negative bacteria—has been implicated in chronic low-grade inflammation via MyD88-mediated pathways, contributing to systemic disorders such as metabolic syndrome and MASLD^89,90^. Other TLRs also participate in host-microbe regulation within the gut, each with unique ligand specificities and functional outputs^91–94^. Despite this expanding knowledge, direct evidence of gut PRRs specifically recognizing oral-derived microorganisms or their components remains limited. Future studies are needed to clarify the spatial and functional specificity of PRR-mediated recognition of translocated oral microbes, especially in the context of mucosal immune reprogramming and systemic inflammation.

## Systemic inflammation initiated from the gut

### Gut barrier disruption and immune activation

The intestinal barrier constitutes the primary defense against microorganisms and toxins present in the gut lumen. Structurally, it consists of an outer mucus layer, a single layer of IECs, and the underlying lamina propria populated with immune cells^95^. Tight junction proteins, such as occludin, claudins, and ZO-1, secure the integrity of IECs and regulate paracellular permeability^96^. Certain oral bacteria produce proteolytic enzymes or express outer membrane components that can disrupt tight junctions^97^, induce epithelial apoptosis, or degrade the mucus layer^98^, thereby promoting the leakage of MAMPs and chronic immune activation.

*P. gingivalis* has been shown to degrade tight junction proteins such as ZO-1 via its gingipain proteases, compromising epithelial integrity and barrier function^99^. Its BEVs, containing the lysine-specific gingipain (Kgp), degrade occludin from the cytoplasmic side and trigger RIPK1-dependent necroptosis in IECs^36,100^. Infection with *F. nucleatum* induces ferroptosis in Caco-2 cells, suppresses cell proliferation, and compromises epithelial barrier integrity by degrading ZO-1 and claudin-1 (CLDN1)^101^. Additionally, the pathogenic *Campylobacter concisus* strain AToCC secretes zonula occludens toxin (Zot), which increases epithelial permeability, drives inflammation, and induces caspase 3/7-dependent apoptosis in IECs^102,103^. *Klebsiella pneumoniae* disrupts IECs through a transcellular route, involving activation of Rho GTPase and PI3K/Akt signaling pathways and cytoskeletal rearrangement^104^.

When the epithelial barrier is compromised, MAMPs can more readily enter the systemic circulation, eliciting both local and systemic immune activation^105^. For example, *P. gingivalis* exacerbates neutrophil infiltration and increases proinflammatory cytokines—including IL-1β, IL-6, and TNF-α—in colitic mice^99^. *F. nucleatum*-derived BEVs promote polarization of proinflammatory M1 macrophages via TLR4 signaling, upregulating TNF-α, IFN-γ, and IL-6 while downregulating IL-10^36,106^. *P. gingivalis* infection also increases Th17 cells and decreases Tregs, accompanied by a rise in IL-9⁺CD4⁺ T cells in the small intestinal lamina propria^107,108^. IL-9, a pleiotropic proinflammatory cytokine, plays a key role in IBD, where it promotes pathogenesis via the miR-21–CLDN8 pathway, suggesting that this axis is crucial for regulating intestinal barrier function. Moreover, these downstream immunological consequences appear to be contingent upon a pre-existing inflammatory or barrier-disrupted intestinal milieu. In models of chronic periodontitis, oral overgrowth of *Klebsiella* and *Enterobacter* induces Th17 polarization in the oral mucosa, followed by migration to the gut and exacerbation of intestinal inflammation^109^. Similarly, oral administration of *K. pneumoniae* promotes intestinal Th1 responses, activates TLR4 in gut-resident dendritic cells, and induces IL-18 secretion, thereby further amplifying mucosal immune activation^85,110^.

## Potential of cross-organ immune communication

Oral microbes can disrupt the intestinal barrier, allowing MAMPs to enter the systemic circulation, where they trigger widespread immune responses and organ-specific inflammatory cascades. Beyond acute, cytokine-driven activation, emerging evidence indicates that oral microbes can orchestrate cross-organ immune communication, involving both innate and adaptive immune compartments. This process encompasses the activation, migration, and functional modulation of immune cells across distant tissues^111,112^.

Under steady-state conditions, leukocytes originating from cervical lymph nodes (CLNs) can migrate to the gut^113^. During oral inflammation, Th17 cells activated by oral pathogens can home to the gut and undergo reactivation, exacerbating intestinal inflammation. Notably, this reactivation appears to be initiated by innate immune cues, particularly gut mucosal IL-1β induced by oral pathobionts. This effect has been validated in macrophages, which secrete IL-1β in response to the oral strain, subsequently promoting the activation and functional expansion of transmigrated T cells. These findings highlight that oral microbes first engage innate immune mechanisms, which then shape downstream adaptive immune responses in distal sites^109,112^.

While numerous studies support a link between local immune stimulation and distal immune communication^114^, alternative models propose “regional immune specialization,” whereby distinct mucosal tissues maintain compartmentalized immune sensing and homing programs. For instance, one study showed that mandibular lymph nodes (mandLNs) act as sentinel sites that efficiently capture *Listeria monocytogenes* upon oral ingestion and initiate early CD8⁺ T cell responses. However, unlike T cells activated in mesenteric lymph nodes (MLNs) during intestinal exposure, mandLN-derived effector T cells lack canonical gut-homing markers^115^. These findings suggest that oral immune stimulation does not universally induce cross-mucosal effects, and whether it can elicit immune communication with functional consequences in distal sites such as the gut remains to be elucidated through targeted experimental models.

Together, these observations indicate that the extent of cross-organ immune communication depends on the tissue of immune activation and the migratory programming of the responding cells.

## Beyond the gut: systemic implications

Although the gut mucosa is the primary interface for host–microbe interactions, the immunological impact of oral bacterial translocation extends beyond the intestine. Emerging evidence indicates that the oral–gut axis can influence distal organs, including the liver and central nervous system (CNS), creating a complex network of inter-organ immune communication.

The liver is anatomically and functionally connected to the gut via the portal vein, receiving approximately 70–75% of its blood supply from the gastrointestinal tract^116^. This vascular connection allows continuous delivery of dietary nutrients, microbial metabolites, and translocated microbial products to the liver^117,118^. Dysbiosis in the oral and gut microbiota has been increasingly linked to liver disease pathogenesis. Oral microbes can disrupt gut microbial homeostasis, compromise epithelial barrier integrity, and promote endotoxemia. This facilitates the translocation of microbial components and metabolites to the liver through the portal circulation, initiating hepatic inflammation and immune dysregulation^119,120^. Experimental studies have shown that oral administration of *P. gingivalis* or *F. nucleatum* in mice induces gut dysbiosis, increases intestinal permeability, elevates systemic LPS, and causes hepatic steatosis, resembling MASLD^121,122^. Once in the liver, microbial MAMPs transported via the portal vein are detected by liver sinusoidal endothelial cells, Kupffer cells, and other resident immune cells through TLRs. This recognition triggers the release of pro-inflammatory cytokines, including TNF-α and IL-1β^123^. Microbial metabolites, including LPS and butyrate, influence the Th17/Treg balance, thereby affecting hepatic immune homeostasis, inflammation, and hepatocellular injury^124,125^. LPS exposure skews this balance toward Th17 dominance and Treg reduction, weakening immune tolerance and aggravating liver injury^126^. During MASLD progression, IL-17 serves as a key effector cytokine: it impairs insulin signaling in hepatocytes, synergizes with fatty acids to promote steatosis, and activates Kupffer and stellate cells, amplifying IL-6/TGF-β–mediated inflammatory and fibrotic pathways. This establishes a Th17–IL-17–IL-6 feedback loop, driving the transition from metabolic disturbance to chronic hepatic inflammation and fibrosis^124^.

LPS also induces hepatocyte apoptosis by upregulating TNF-α, sustaining low-grade p38 MAPK activation, and promoting neutrophil infiltration and MPO release, which collectively aggravate cellular injury^127,128^. The resulting apoptotic hepatocytes are engulfed by macrophages, triggering the release of proinflammatory mediators that activate stellate cells and accelerate collagen deposition and fibrogenesis^129^. Conversely, the gut microbial metabolite butyrate exerts hepatoprotective effects by restoring Th17/Treg balance and attenuating fibrotic signaling. It activates PPARγ and reprograms energy metabolism to promote Treg differentiation while suppressing Th17 polarization^130^. Butyrate also alleviates MCD-diet–induced fibrosis by downregulating TGF-β1 and α-SMA, enhancing intestinal barrier integrity, and reducing endotoxin levels^131^. Clinical evidence from cirrhotic patients reinforces this axis, revealing enrichment of oral-origin taxa in the intestinal microbiome^132^ and demonstrating that periodontal treatment can reduce systemic inflammation^133^. Specifically, cirrhotic patients display increased *Veillonellaceae* and *Lactobacillaceae* in saliva and a similar “oralization” pattern in feces. Following periodontal therapy, serum endotoxin, LBP, and IL-6 levels decrease, while beneficial gut taxa such as *Ruminococcaceae* increase, and oral-derived taxa (*Porphyromonadaceae*, *Streptococcaceae*) decline. In contrast, untreated cirrhotic patients show rising endotoxin and LBP levels over the same period. These findings highlight that modulation of the oral environment can beneficially reshape gut microbiota and systemic inflammation, reinforcing the clinical relevance of the oral–gut axis in liver disease progression^133^.

The CNS is also susceptible to microbial influence through the oral–gut–brain axis. The gut–brain axis is known to regulate CNS function via neural, immune, endocrine, and metabolic pathways. Expanding on this concept, oral microbiota may influence brain immune responses and neurophysiology, particularly in neurodegenerative disorders such as cognitive impairment, AD, and PD^134^. Epidemiological studies have linked chronic oral inflammation, particularly periodontitis, to cognitive decline and elevated risk of AD^135^. Patients with periodontitis often show elevated pro-inflammatory cytokines (e.g., IL-6, TNF-α) in cerebrospinal fluid, indicating that systemic inflammation triggered by oral infection may disrupt CNS homeostasis^136,137^. Furthermore, *P. gingivalis* and its virulence factors, such as gingipains, have been identified in the brains of AD patients, supporting the hypothesis of microbial translocation across biological barriers into the CNS^138^. Mechanistically, oral bacterial colonization of the gut can compromise the intestinal barrier, leading to dysbiosis and triggering neuroimmune responses. Barrier disruption activates macrophages and T cells, which release inflammatory mediators that reach the brain via hematogenous or vagal pathways, ultimately inducing neuroinflammation^139^. In AD mouse models, oral pathogens disrupt gut integrity, induce hippocampal inflammation, enhance amyloid-β (Aβ) deposition, and worsen cognitive performance^140,141^. At the metabolic level, gut-derived short-chain fatty acids (SCFAs), such as butyrate and propionate, regulate CNS and peripheral nervous system (PNS) activity via G protein–coupled receptors. Certain SCFAs, such as acetate, propionate, and butyrate, can cross the blood–brain barrier and modulate neuronal plasticity, neurotransmitter biosynthesis, and neuroimmune signaling^142–145^. Acetate, propionate, and butyrate also influence gene expression via epigenetic mechanisms, including histone deacetylase (HDAC) inhibition, thus offering a molecular link between microbiota and neuronal function^146,147^. Dysbiosis caused by oral microbes may impair SCFA production^148^. For example, in periodontitis mouse models, significant changes were observed in the gut, including a reduction in SCFA-producing bacteria such as *Lactobacillus*, *Ligilactobacillus*, and *Allobaculum*, as well as a notable decrease in *Firmicutes* and *Actinobacteriota*^149^. The reduction in SCFAs may compromise intestinal barrier integrity and amplify systemic inflammation, ultimately leading to increased soluble Aβ, phosphorylated tau, amyloid plaques, neurofibrillary tangles (NFTs), and persistent systemic neuroinflammation^150^. Additionally, certain microbes can directly affect neuronal signaling by producing neuroactive compounds such as γ-aminobutyric acid (GABA), serotonin, dopamine, and acetylcholine, which influence synaptic function and behavior^151^. Neural circuits further enhance this communication. The vagus nerve serves as a major bidirectional conduit between the gut and brain, transmitting mechanical, metabolic, and inflammatory signals to the brainstem, particularly the nucleus tractus solitarius (NTS)^152,153^. Through dense vagal innervation, the gut–brain axis integrates CNS and intestinal immune signals, with the vagus nerve playing a central role in anti-inflammatory signaling and neuroendocrine modulation^154^. In response to gut dysbiosis or inflammation, vagal activation stimulates the hypothalamic–pituitary–adrenal axis, affecting mood and cognition^155,156^. Notably, vagotomy abrogates the antidepressant effects of microbiota-based therapies, underscoring the essential role of the vagus nerve in microbiota–brain communication^157^.

In summary, oral microbiota modulate CNS immune function and neural activity via gut colonization, barrier disruption, metabolic reprogramming, and neural signaling, providing a novel mechanistic perspective on brain disorder pathogenesis.

## Clinical implications and interventional strategies

As the pathogenic significance of the oral–gut axis in chronic multi-organ diseases becomes increasingly evident, the development of effective interventional strategies has emerged as a key focus in translational medicine. Current studies and clinical practices have begun to explore multi-tiered approaches targeting various stages of this axis—from inhibiting oral microbial dissemination, modulating the intestinal colonization niches to restoring gut barrier integrity and mitigating systemic immune activation. These strategies directly address the oral microbial translocation and systemic immune activation described above.

### Inhibition of oral microbial migration to the gut

Periodontal pathogens can reach the gastrointestinal tract via many routes mentioned above, where they disrupt the gut microbiome and aggravate local inflammation. Restricting cross-regional microbial translocation thus represents a critical upstream strategy to contain their systemic pathogenicity. Basic periodontal therapy, such as scaling and root planing, significantly reduces oral pathobiont burden. In human studies, genera such as *Porphyromonas*, *Saccharimonas*, and *Selenomonas* became undetectable in gut samples post-treatment, while *Faecalibacterium* levels were restored to those of healthy controls^158^. This intervention also lowers circulating LPS levels^133^ and reduces systemic inflammatory markers^159,160^ (e.g., CRP, IL-6, TNF-α), which may indirectly alleviate inflammation-induced impairment of the intestinal barrier. Similarly, in mouse models, non-surgical periodontal treatment partially restores intestinal barrier integrity—including villus height and mucosal injury scores—and normalizes gut microbiota composition^161^.

AMPs, which exert both direct bactericidal and immunomodulatory effects, have also been explored to rebalance the oral–gut microbiota. Oral administration of AMPs such as LL-37 and M33D suppresses biofilm formation and inhibits adhesion and invasion of migratory oral pathogens^162–164^. In addition, AMP R71 enhances intestinal mucosal immunity by promoting secretory IgA production, stabilizing tight junctions, and downregulating TLR4-mediated inflammatory responses^165^. Some AMPs also silence virulence genes in oral bacteria, reducing pathogenicity at the molecular level^166^.

Photobiomodulation (PBM), a non-invasive light-based technique, has emerged as a novel tool to reshape oral microbial ecology. PBM can directly act on microbial photoreceptors such as cytochromes, flavoproteins, and ferritins via photo-energization, thereby altering microbial metabolism and viability^167^. It also modulates the host response: pretreatment of human gingival keratinocytes with PBM (red: 615 nm; near-infrared: 880 nm) induces AMP production and downregulates pro-inflammatory activity^168^. In vitro studies of interdental plaque exposed to violet LED irradiation demonstrated reduced total bacterial load, altered microbial diversity (α- and β-diversity), and selective inhibition of pathogenic genera such as *Fusobacterium* and *Prevotella*. Together, these effects indicate that PBM can reshape the oral microbiome both directly and indirectly by modulating the host microenvironment^169^.

Collectively, these strategies—from periodontal therapy to AMPs and PBM—contribute to inhibiting oral microbial migration to the gut.

### Modulation of the intestinal environment

Even when oral microbes reach the intestine, their pathogenic potential depends largely on the local gut milieu. Therefore, modulating gut microbiota composition and restoring barrier function represent critical midstream strategies to prevent downstream immune activation.

Probiotics such as *Lactobacillus rhamnosus* GG and *Bifidobacterium infantis* exert multifaceted benefits, including competitive exclusion of pathogens, lactic acid production, and tight junction reinforcement. They also produce biosurfactants and antimicrobial compounds, which inhibit colonization and proliferation of oral-derived strains in the gut^170^. Specifically, *Bifidobacterium* BB-12 and *Lactobacillus rhamnosus* GG help preserve intestinal barrier integrity and stabilize gut microbiota. They modulate inflammatory signaling via competitive inhibition of pathogen adhesion, activation of host immune responses, clearance of toxins, and production of bacteriocins, H₂O₂, and organic acids. In neuroinflammatory models such as AlCl₃-induced AD, these probiotics transiently colonize the gut, reduce pro-inflammatory cytokines (TNF-α, IL-1β), attenuate neuroinflammation, and modulate neurotransmitter signaling (e.g., GABA receptor expression), ultimately improving cognition after 28 days of administration^171^.

Oral microbial colonization is frequently associated with loss of SCFA-producing taxa, such as *Bacteroidota* and *Prevotellaceae*. Supplementation with dietary fiber or SCFA precursors enhances microbial fermentation and SCFA production, thereby restoring gut homeostasis^172^. SCFAs activate GPCR41/43 or inhibit HDACs. These actions enhance the expression of tight junction proteins ZO-1 and occludin, suppress NLRP3 inflammasome activation, and reduce IL-1β and IL-6 levels^173–176^.

In summary, gut-targeted modulation serves both as an ecological barrier against oral microbial colonization and as a central strategy to prevent immune spillover into distal organs.

## Future perspectives

As the pathophysiological relevance of the oral–gut axis in systemic inflammation, immune reprogramming, and multi-organ chronic diseases becomes increasingly evident, single-dimensional approaches are no longer sufficient to support in-depth mechanistic exploration or precision intervention. Recent advances in multi-omics technologies, organ-on-a-chip models, and functional microbial databases have begun to offer systematic solutions for decoding the complex interplay between oral microbes, the gut, and distal organ systems.

### Multi-omics integration

The processes by which oral microbes colonize the gut, modulate local immune responses, disrupt metabolic homeostasis, and influence distant organs involve complex, multilayered mechanisms. Integrated multi-omics approaches have emerged as a powerful toolkit for deciphering these networks, enabling simultaneous interrogation of microbial taxa, host immune mediators, and metabolic pathways. By combining metagenomics, transcriptomics, proteomics, metabolomics, and single-cell omics, researchers can generate high-resolution maps that link specific oral taxa with systemic disease phenotypes^177^.

However, challenges remain, including data heterogeneity, a lack of standardized analytical algorithms, and the difficulty of biological interpretation. To overcome these barriers, artificial intelligence and machine learning algorithms should be employed to enhance integration and identify nonlinear patterns. Moreover, leveraging large-scale cohort datasets and real-world microbiome data will facilitate risk stratification and personalized disease prediction in oral–gut-related disorders.

### Organ-on-a-chip models simulating the “oral–gut–organ axis”

Conventional animal and 2D cell models often fail to recapitulate the multiorgan crosstalk and dynamic signaling involved in the oral–gut axis. The emergence of organ-on-a-chip platforms has introduced a transformative approach to model this axis in vitro under physiologically relevant conditions. These microfluidic, 3D-engineered systems combine human-derived cells, tissue-mimetic scaffolds, and biomechanical cues to replicate architecture and function at the organ level^178^. Compared with animal models, organ chips offer superior predictive fidelity for human physiology and drug response, while significantly reducing preclinical failure rates^179^.

Notably, several organ-on-a-chip platforms have been developed to mimic the structural and microbial features of oral tissues. Gum-on-a-chip is a microfluidic organ-on-chip platform designed to recapitulate the physiological microenvironment of human gingival tissue. These models typically integrate a triculture of keratinocytes, fibroblasts, and endothelial cells, forming a 3D mucosal barrier for studying gingival inflammation and therapeutic interventions^180^. Gingiva-on-chip employs a vertically stacked microfluidic architecture to generate full-thickness gingival equivalents. This design supports long-term air–liquid interface culture and dynamic flow conditions. Compared with static cultures, it enhances epithelial morphogenesis and barrier stability, enabling modeling of both healthy and diseased states (e.g., ulcer-like barrier disruption)^181^. Gingival crevice-on-a-chip models focus on the microenvironment of the gingival crevice, emphasizing dynamic gingival crevicular fluid flow. This system reproduces interactions between oral mucosa and bacteria; for example, exposure to *Streptococcus mutans* decreases transepithelial electrical resistance, reflecting bacterial-induced barrier dysfunction^182^.

Gut-on-a-chip models co-culturing intestinal microbes have proven effective for simulating intestinal pathophysiology^183^. More complex organ chips—such as gut-liver^184^ and gut-skin^185^—are also being explored. Developing integrated oral–gut–organ chips may therefore provide novel insights into immune-mediated diseases involving the oral–gut axis.

### Construction of microbe–function–immune response databases

To understand how oral microbes affect gut physiology and systemic diseases via colonization, functional activity, and immune signaling, it is essential to systematically map microbial identities to their host impact profiles. Development of structured databases that annotate “microbe-function-immune response” interactions will serve as a cornerstone for translational microbiome research.

Although existing resources such as FAPROTAX^186^ and MetaCyc^187^ provide microbial functional annotations, they still lack a direct link to host immune phenotypes. Emerging platforms like GIMICA^188^ (linking host immunity with microbiota) and Cancer-mbQTL^189^ (connecting microbial traits with tumor immune infiltration) offer an early framework to bridge this gap.

Such resources could support development of colonization-potential scores or immune perturbation indices for oral taxa, predicting their likelihood of engrafting and triggering immune dysregulation in distant organs. These tools will accelerate the translation of microbiome science into precision diagnostics and targeted therapeutics in clinical immunology and infectious diseases.

## Conclusion

Oral microbiota can translocate beyond the oral cavity, disrupt gut homeostasis, and influence systemic immune responses through the oral-gut-organ axis. This review highlights the key mechanisms by which oral microbes affect barrier integrity, immune signaling, and chronic inflammation in distal organs such as the liver and brain. Notably, their ability to induce trained immunity may contribute to persistent immune dysregulation. Current interventional strategies—including AMPs, probiotics, and gut-targeted metabolic modulation—offer promising avenues, but further research integrating organ-on-a-chip models and multi-omics approaches is needed to advance clinical translation.

## Data Availability

No datasets were generated or analysed during the current study.
